# The Current Recommended Vitamin D Intake Guideline for Diet and Supplements During Pregnancy Is Not Adequate to Achieve Vitamin D Sufficiency for Most Pregnant Women

**DOI:** 10.1371/journal.pone.0157262

**Published:** 2016-07-01

**Authors:** Fariba Aghajafari, Catherine J. Field, Bonnie J. Kaplan, Doreen M. Rabi, Jack A. Maggiore, Maeve O’Beirne, David A. Hanley, Misha Eliasziw, Deborah Dewey, Amy Weinberg, Sue J. Ross, APrON Study Team

**Affiliations:** 1 Cumming School of Medicine, University of Calgary, Calgary, Alberta, Canada; 2 Department of Agricultural, Food and Nutritional Science, University of Alberta, Edmonton, Alberta, Canada; 3 Research and Development, Doctor’s Data, Inc., St. Charles, Illinois, United States of America; 4 Department of Public Health and Community Medicine, Tufts University, Boston, Massachusetts, United States of America; 5 Department of Obstetrics and Gynecology, University of Alberta, Edmonton, Alberta, Canada; University of Queensland, AUSTRALIA

## Abstract

**Background:**

The aims of this study were to determine if pregnant women consumed the recommended vitamin D through diet alone or through diet and supplements, and if they achieved the current reference range vitamin D status when their reported dietary intake met the current recommendations.

**Methods:**

Data and banked blood samples collected in second trimester from a subset of 537 women in the APrON (Alberta Pregnant Outcomes and Nutrition) study cohort were examined. Frozen collected plasma were assayed using LC-MS/MS (liquid chromatography-tandem mass spectrometry) to determine 25(OH)D_2_, 25(OH)D_3_, 3-epi-25(OH)D_3_ concentrations. Dietary data were obtained from questionnaires including a Supplement Intake Questionnaire and a 24-hour recall of the previous day’s diet.

**Results:**

Participants were 87% Caucasian; mean (SD) age of 31.3 (4.3); BMI 25.8 (4.7); 58% were primiparous; 90% had education beyond high school; 80% had a family income higher than CAN $70,000/year. 25(OH)D_2_, 25(OH)D_3_, and 3-epi-25(OH)D_3_) were identified in all of the 537 plasma samples;3-epi-25(OH)D_3_ contributed 5% of the total vitamin D. The median (IQR) total 25(OH)D (D_2_+D_3_) was 92.7 (30.4) nmol/L and 20% of women had 25(OH)D concentration < 75 nmol/L. The median (IQR) reported vitamin D intake from diet and supplements was 600 (472) IU/day. There was a significant relationship between maternal reported dietary vitamin D intake (diet and supplement) and 25(OH)D and 3-epi-25(OH)D_3_ concentrations in an adjusted linear regression model.

**Conclusions:**

We demonstrated the current RDA (600 IU/ day) may not be adequate to achieve vitamin D status >75 nmol/L in some pregnant women who are residing in higher latitudes (Calgary, 51°N) in Alberta, Canada and the current vitamin D recommendations for Canadian pregnant women need to be re-evaluated.

## Introduction

Vitamin D plays an important role in promoting healthy pregnancy and fetal development, and poor vitamin D status has been associated with adverse maternal and infant health [1]. There is also evidence that pregnancy risks may be mitigated by vitamin D supplementation, although the optimal daily dose is not clear [2]. Health Canada [3] and the Institute of Medicine (IOM) [4] have recommended 600 IU/day intake of vitamin D during pregnancy. The IOM set these guidelines on optimizing bone health of pregnant women (and ensuring bone growth in the infant) [4]. Currently, there is a lack of consensus on what constitutes vitamin D sufficiency. The IOM has recommended a 25(OH)D cut point of >50 nmol/L to define vitamin D sufficiency, as this level is associated with prevention of the bone manifestations of vitamin D deficiency (rickets and osteomalacia) for 97.5% of Canadians and Americans. However, there is not universal agreement with the IOM recommendations, and others suggest that an optimal level for bone health might be somewhat higher. Osteoporosis Canada [5] and the Endocrine Society [6] set a serum concentration of >75 nmol/L as the target for optimal bone health, and other experts have suggested concentrations > 80 nmol/L [7].

Despite these recommendations, a recent study showed one-quarter of Canadians (ages 6–79 years) did not meet the Recommended Daily Allowance (RDA; daily intake level of a nutrient that is considered to be sufficient to meet the requirements of 97–98% of healthy individuals) through food sources alone. However, the daily use of vitamin D supplements contributed to a better 25-hydroxycholecalciferol (25(OH)D) status [8]. A combined analysis of two randomized clinical trials of 494 pregnant women taking 400, 2000, and 4000 IU/day vitamin D during pregnancy showed that vitamin D supplementation of 4000 IU/day was more effective in achieving serum concentrations ≥ 80 nmol/L than 400 and 2000 IU/day in all women and their neonates regardless of race, suggesting that the current dietary recommendation in the absence of supplementation in pregnancy may not be optimal [9]. However, these studies used sub-optimal quantification methods (radio-immunoassays) and did not consider the potential contribution of dietary vitamin D to total intake. A recent systematic review of vitamin D supplementation in pregnancy concluded that evidence is currently insufficient to support definite clinical recommendations regarding vitamin D supplementation in pregnancy. This review highlighted the extremely heterogeneous evidence in regards to definition of serum vitamin D concentration threshold and the lack of high-quality interventional studies [10].

The best clinical laboratory indicator of vitamin D status is the blood concentration of 25(OH)D [4]. It is important to use an assay methodology that can identify both 25-hydroxyergocalciferol (25(OH)D_2_) and 25(OH)D_3_, as both metabolites are metabolically active and both are available as dietary supplements and in fortified foods. In addition, recent studies have shown the epimeric form of 25(OH)D_3_ contributes significantly to measured 25(OH)D_3_ when methods are unable to separate the less active epimer [11, 12]. We recently showed that not separating out plasma 3-epi-25-hydroxycholecalciferol (3-epi-25(OH)D_3_) from vitamin D status calculation underestimates the classification of pregnant women and their newborns at risk for vitamin D insufficiency (>75 nmol/L) [13].

Although the literature regarding vitamin D in pregnancy is growing rapidly, several gaps in our knowledge remain, including the prevalence of vitamin D deficiency/ insufficiency during pregnancy and the intake of vitamin D during pregnancy needed to ensure optimal status. Our aims in the current study were to determine: a) if pregnant women were consuming the recommended vitamin D through diet alone or through diet and supplements, and b) if pregnant women can achieve the current reference range vitamin D status when their reported dietary intake of vitamin D met the current recommendations.

## Methods

This study is a secondary analysis of an established maternal and infant prospective cohort study. The APrON study (Alberta Pregnancy Outcomes and Nutrition; www.ApronStudy.ca) is a longitudinal cohort of pregnant women and their children residing in Calgary and Edmonton (Alberta, Canada). We used the APrON cohort data and analyzed the banked blood samples collected from the first 537 women recruited to APrON in their second trimester of pregnancy (whose dietary data had been cleaned and linked to the biomedical data). APrON recruited a total of 2191 women between March 2009 and July 2012, primarily through in-person contact at maternity clinics. Full details of the APrON study are described elsewhere [14].

### Maternal dietary data

#### 24-hour recall

At each prenatal and postpartum visit, women were asked by a trained nutrition professional or registered dietitian to describe in detail the quantity and type of both food and beverages consumed in the previous 24-hour period midnight to midnight during each trimester and three months postpartum. Dietary food models were utilized to help increase accuracy of serving size estimates. The trained nutrition professional or registered dietitian also used probing tactics to obtain further information on cooking method, meal times, and food brands, as well as completeness of recall. Dietary recalls were read back to each participant for accuracy and comprehensiveness. Analysis of these records is described in detail elsewhere [15]. The estimated dietary intake of vitamin D from the 24-hour recall was not specifically validated against another dietary intake tool. However, intake from food and beverages only accounted for 39% of total vitamin D intake and the major source (39%) was from milk, which was consumed on a regular basis by participants during the second trimester of pregnancy. The major source for vitamin D was from the supplements; this data was obtained from the questionnaire that has been previously described in detail [15].

#### Maternal Supplement Intake Questionnaire (SIQ)

Following enrollment, the Maternal Supplement Intake Questionnaire (SIQ) was administered by a trained nutrition professional to each participant once during each trimester of pregnancy and once at 3 months postpartum to determine current Natural Health Product (NHP) use including vitamin D. The SIQ was designed specifically for the APrON study and was initially used in a pilot study that included of fifty women in the APrON study who completed the questionnaire during their first and second visits to assess efficacy and the detail of information obtained [15]. Women were encouraged to bring in the supplement bottles they consumed that contain the Natural Product Number (NPN) that is linked to Health Canada’s Licensed Health Products Database (LNHPD) [15]. If women did not bring in the supplement bottles, they reported the brand name, dosage, and frequency of consumption for each product during the period between visits. When a nutritional supplement could not be found in the LNHPD, the manufacturer’s website was used to retrieve nutritional information. Since the SIQ was administered when the women began taking the supplements, those who were recruited at 14–26 weeks were able to provide supplement intake retrospectively for the first trimester. At follow-up visits, if women had begun or stopped supplements since their last visit, these adjustments were also made to their supplement intake information. A NHP database was created for this and other APrON studies that linked the NPN to the nutrient content of each supplement. Supplements not in the LNHPD were provided their own unique code for identification purposes. The most common NHPs were used as a default if the nutrient content of the supplement was unknown or if not enough information was provided about the supplement. A detailed conversion method was developed so as to not overestimate intake if participants had taken a supplement for a fraction of the time point or if they switched supplements at any point during that time period. Corrections were applied based on daily use (numbers of days/ week) and trimester (number of weeks/ trimester). Information on supplement intake recording and analysis for the APrON study has been previously reported [15]. All extreme values of vitamin D (>4000, which is the upper limit recommended intake) were reviewed by reviewing the actual SIQ. All values were correct and it was plausible that women could exceed the recommendation. All NHPs were available through health food shops. No participants took a separate vitamin D supplement through a prescription, although a prenatal supplement would have been recommended by their physician. Calcium intake among women in the APrON cohort has not yet been estimated. However, previous studies have shown calcium source during pregnancy is predominately from diet rather than supplements [16–20]. These studies showed calcium supplement contributed less than 20% to the total intake [16–20].

### Measurement of vitamin D status

Maternal blood was collected at each clinic visit by a certified phlebotomist. The majority of APrON participants were recruited in their second trimester; therefore, we used the blood samples taken from their second trimester for this study (consequently, the dietary data from the second trimester were used for analysis). All frozen collected plasma was assayed alongside plasma quality control samples and standard reference materials using a clinically validated LC-MS/MS assay at the laboratory of Doctor’s Data Inc. [13], which measured 25(OH)D_2_, 25(OH)D_3_, and 3-epi-25(OH)D_3_ and demonstrates an intra-assay coefficient of variability of 4.6% at 28.3 nmol/L, 3.3% at 83.9 nmol/L, and 3.9% at 5.8 nmol/L for 25(OH)D_2_, 25(OH)D_3_, and 3-epi-25(OH)D_3_, respectively. Information on processing blood samples and integrity of plasma samples has been previously reported [13]. The concentrations of 25(OH)D_2,_ 25(OH)D_3,_ and 3-epi-25(OH)D_3_ in plasma were determined using LC-MS/MS (the National Institute of Standard and Technology (NIST) standards and Vitamin D External Quality Assessment Scheme (DEQAS) procedures were followed), as previously described [13].

### Statistical analysis

Results are presented for plasma 25(OH)D_2_, 25(OH)D_3,_ and 3-epi-25(OH)D_3_ in terms of means and standard deviations (SD) and medians and interquartile ranges (IQR). The prevalence rates of vitamin D deficiency and insufficiency in women were estimated as percentages with 95% confidence interval (CI). Plasma concentrations of 25(OH)D at <25 nmol/L [3, 4], <50 nmol/L [3, 4], and <75 nmol/L [5, 6] were used to reflect several laboratory definitions of vitamin D status currently used. The prevalence rates of vitamin D insufficiency with and without the epimer were compared using McNemar’s chi-square test. The correlation between maternal 25(OH)D_3_ and 3-epi-25(OH)D_3_ was determined using the Pearson correlation coefficient test. Repeated measures ANOVAs were used to examine the difference between the reported dietary intakes across the three trimesters. As reported dietary and supplemental vitamin D intakes were not normally distributed, the natural log transformation of the skewed variables was used for the test.

A multiple linear regression analysis was used to examine the association between women’s plasma 25(OH)D (D_2_ + D_3_) concentration and their reported dietary vitamin D intake in supplements and diet adjusted for the potential covariates of maternal age, maternal BMI at second trimester, season (summer from May 01-Oct 31 and winter from Nov 01-Apr 30), and race (Caucasian *vs*. non-Caucasian) [7]. Again, as the dependent variables (dietary and supplemental vitamin D intakes) and independent variables (25(OH)D_3_ and 3-epi-25(OH)D_3_) were not normally distributed, the natural log transformation of the skewed variables was used for the test.

In addition, plasma levels were dichotomized as deficient/insufficient versus adequate, and multiple logistic regression analysis was used to estimate the odds of insufficiency (<50 *vs*. ≥ 50 and <75 *vs*. ≥ 75 nmol/L) as a function of reported vitamin D intake, adjusted for the same covariates (odds ratios and their corresponding 95% CIs). All analyses were conducted using Stata statistical software (version 14) and SPSS (version 22). P-values < 0.05 were considered statistically significant.

## Results

Blood samples from 537 women from their second trimester of pregnancy who had complete dietary and clinical data were analyzed (sample characteristics in Table 1). In addition, we compared plasma samples from the first and second trimester of 83 APrON participants who were recruited in the first trimester. We measured vitamin D metabolites with LC-MS/MS and showed that, except for 25(OH)D_2_, there were no significant differences between the two trimesters for any other metabolite (Table 2).

**Table 1:** Characteristics of Participants in a Longitudinal Cohort of Pregnant Women in Alberta, Canada.

Characteristics of Participants	Overall (n = 537)
Age, y	31 ± 4 (n = 537)
Gestational age^a^	19.6 ± 3.4 (n = 502)
**Race, %**	(n = 513)
Black	1
Caucasian	87
Others	12
**Family annual income**^b^**, %**	(n = 537)
< $70,000	19
≥ $70,000	81
**Type of education, %**	(n = 514)
< High school	2
High school	8
Trade school	20
Undergraduate	47
Postgraduate	23
**Prior births ≥37 weeks gestation, %**	(n = 537)
0	58
1	32
2	8
3	1
4	0.4
**Season of sampling, %**	(n = 546)
Spring^c^	33
Summer^d^	25
Fall^e^	14
Winter^f^	28
BMI^g^	26 ± 5 (n = 537)

^a^At time of blood sample.

^b^Canadian dollars.

^c^March 20-June 20.

^d^June 21-September 20.

^e^September 21-December 20.

^f^December 21-March 19.

^g^Second trimester.

**Table 2:** Distribution of Plasma Vitamin D Metabolites in the First and Second Trimesters of Pregnancy in a Longitudinal Cohort of Pregnant Women and Their Infants in Alberta, Canada.

Trimesters of Pregnancy	N	Range (nmol/L)	Mean ± SD (nmol/L)	Median (25^th^ to 75^th^) (nmol/L)
**First trimester**				
Total 25(OH)D	83	40.1–161.1	93.3 (25.6)	94.1 (75.4–108.8)
Total 25(OH)D_3_	83	40.1–161.2	92.2 (25.6)	93.3 (73.8 to 108.2)
Total 25(OH)D_2_	83	0–6	1 (1.2)	0.6 (0 to 1.7)
Total 3-epi-25(OH)D_3_	83	2.1–14.7	5.4 (2)	5.0 (4.1 to 6.5)
**Second trimester**				
Total 25(OH)D	537	27.1–196.8	95.3 (25)	92.7 (79 to 109.4)
Total 25(OH)D_3_	537	26.1–194.3	91.9 (25)	89.2 (74.7 to 106)
Total 25(OH)D_2_	537	0–21.2	3.4 (2.9)	2.9 (1.7 to 4.4)
Total 3-epi-25(OH)D_3_	537	0.7–19.4	5.6 (2.2)	5.2 (4.1 to 6.7)
Total 25(OH)D_+_3-epi-25(OH)D_3_	537	29.7–214.3	100.9 (26.6)	98.1 (83.4 to 116.2)

The paired t-test showed a significant difference between first and second trimester for total 25(OH)D_2_ (P<0.0001), but no significant difference for total 25(OH)D_3_ (P = 0.52), total 25(OH)D (P = 0.46), and total 3-epi-25(OH)D_3_ (P = 0.13).

**Reported dietary vitamin D intake (diet and supplements)**. Table 3 shows the reported intake from diet and supplements for all the trimesters of pregnancy. There were 537 completed SIQs and 528 completed 24-hour recalls from the second trimester, and 526 participants provided complete data from both diet and supplements. The median (IQR) overall reported intake of vitamin D (IU) from the combined (24-hour recall + SIQ) was 568 (404) for the first trimester, 600 (472) for the second trimester, and 634 (516) for the third trimester (Table 3).

**Table 3:** Distribution of Reported Dietary Vitamin D Intake (IU/day) in the First, Second, and Third Trimesters of Pregnancy in a Longitudinal Cohort of Pregnant Women and Their Infants in Alberta, Canada.

Trimester	N	Mean ± SD	Min to Max	Median (25^th^ to 75^th^)
**First trimester**				
Supplement intake	398	451 ± 530	0 to 4250	320 (200 to 400)
Dietary intakes	113	185 ± 146	5 to 840	165 (71 to 264)
Overall intakes^a^	112	704 ± 554	32 to 4482	568 (407 to 810)
**Second trimester**				
Supplement intake	537	590 ± 690	0 to 8400	400 (250 to 544)
Dietary intakes	528	218 ± 173	0 to 1350	175 (98 to 298)
Overall intakes^a^	526	812 ± 722	38 to 9209	600 (449 to 921)
**Third trimester**				
Supplement intake	493	634 ± 680	0 to 5427	400 (250 to 689)
Dietary intakes	471	236 ± 193	0 to 1633	192 (105 to 321)
Overall intakes^a^	469	872 ± 695	28 to 3790	634 (469 to 985)

^a^Overall intakes: supplements + diet; the repeated measures ANOVA showed no significant difference in log transformed mean vitamin D intake from diet (24 hour recall) (P = 0.13), but a significant difference in log transformed mean intake from supplement across all trimesters (P<0.001), resulting in a significant difference in total vitamin D intake across all trimesters (P = 0.004).

A repeated measures ANOVA showed no significant difference in log transformed mean reported vitamin D intake from diet (24-hour recall) (P = 0.13) between time-points. However, there was a significant difference in log transformed mean reported intake from supplements between time points (first *vs*. second *vs*. third) (P<0.001), resulting in a significant increase in total reported vitamin D intake from trimester one to trimester three (P = 0.004). Fifty-four percent of pregnant women reported taking 600 and higher IU/day of vitamin D through their diet and supplements in the second trimester of pregnancy; maternal characteristics were not different between those who reported taking <600 IU/day vitamin D *vs*. ≥600 IU/day, except university degree (67% *vs*. 78%, respectively) (data are not shown).

**Fig 1:**
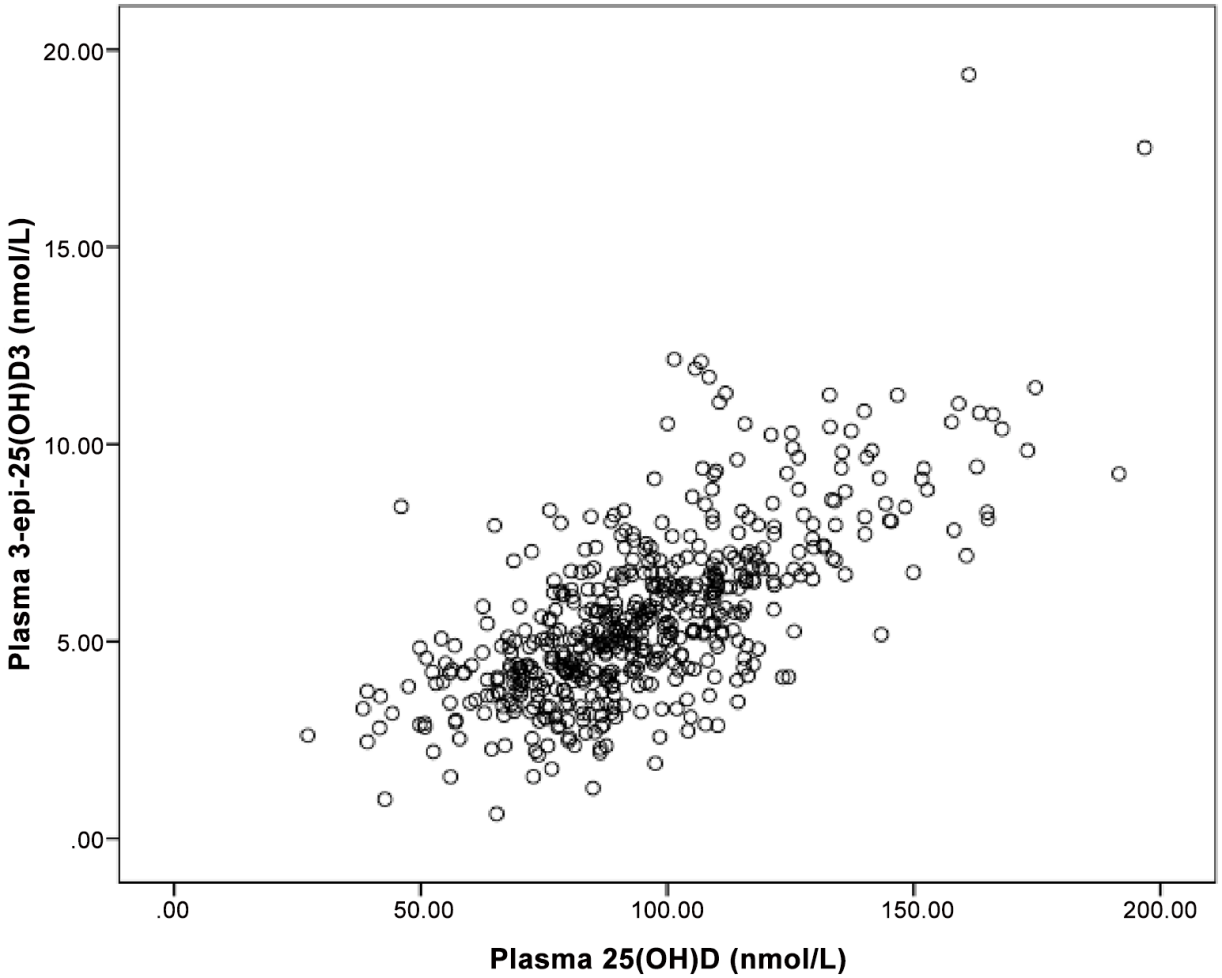
Scatter plot of plasma 3-epi-25(OH)D_3_ and 25(OH)D_3_ in pregnant women during second trimester of pregnancy in a longitudinal cohort of pregnant women and their infants in Alberta, Canada (n = 537). Pearson correlation coefficient showed a significant correlation between 25(OH)D_3_ and 3-epi-25(OH)D_3_ (r = 0.69, P<0.001).

**Maternal 25(OH)D**_**2**_**, 25(OH)D**_**3**_ **and 3-epi-25(OH)D**_**3**_ **concentrations**. All samples were found to have detectable concentrations of 25(OH)D_2_, 25(OH)D_3,_ and 3-epi-25(OH)D_3_. Median (25^th^-75^th^) maternal plasma concentration for 25(OH)D_2_ was 2.9 (1.7–4.4) nmol/L. Vitamin D supplements in Canada are almost all vitamin D_3_; however, vitamin D_2_ is still available by prescription and may be obtained from some food sources. Median (25^th^-75^th^) maternal plasma 25(OH)D_3_ was 89.2 (74.4–106) nmol/L, and for plasma 3-epi-25(OH)D_3_ was 5.2 (4.1–6.7) nmol/L (Table 2). No participant had 25(OH)D (excluding 3-epi-25(OH)D_3_) plasma concentrations <25 nmol/L, 12 (2%, 95% CI: 1.1–3.5) women had plasma concentrations <50 nmol/L, and 100 (19%, 95% CI: 15.3–22.2) women had concentrations in the <75 nmol/L range. The relationship between 25(OH)D_3_ and 3-epi-25(OH)D_3_ is shown in Fig 1.

**The effect of 3-epi-25(OH)D**_**3**_ **on the classification of 25(OH)D sufficiency**. Three-epi-25(OH)D_3_ was detected (>1.0 nmol/L) in all maternal samples. Three-epi-25(OH)D_3_ comprised 5.0% of total 25(OH)D. When 3-epi-25(OH)D_3_ was excluded from the vitamin D estimations, 19% of women were considered to have levels <75 nmol/L; however, with 3-epi-25(OH)D_3_ included, the percentage of women with vitamin D concentration of <75 nmol/L decreased to 12%. The observed difference in the risk of insufficiency was significant (P<0.001). As we had very small numbers of participants with 25(OH)D concentrations <50 nmol/L, the estimate of insufficiency at this level with 3-epi-25(OH)D_3_ excluded was 2%, compared to 1.5% when it was included (P = 0.125).

**Fig 2:**
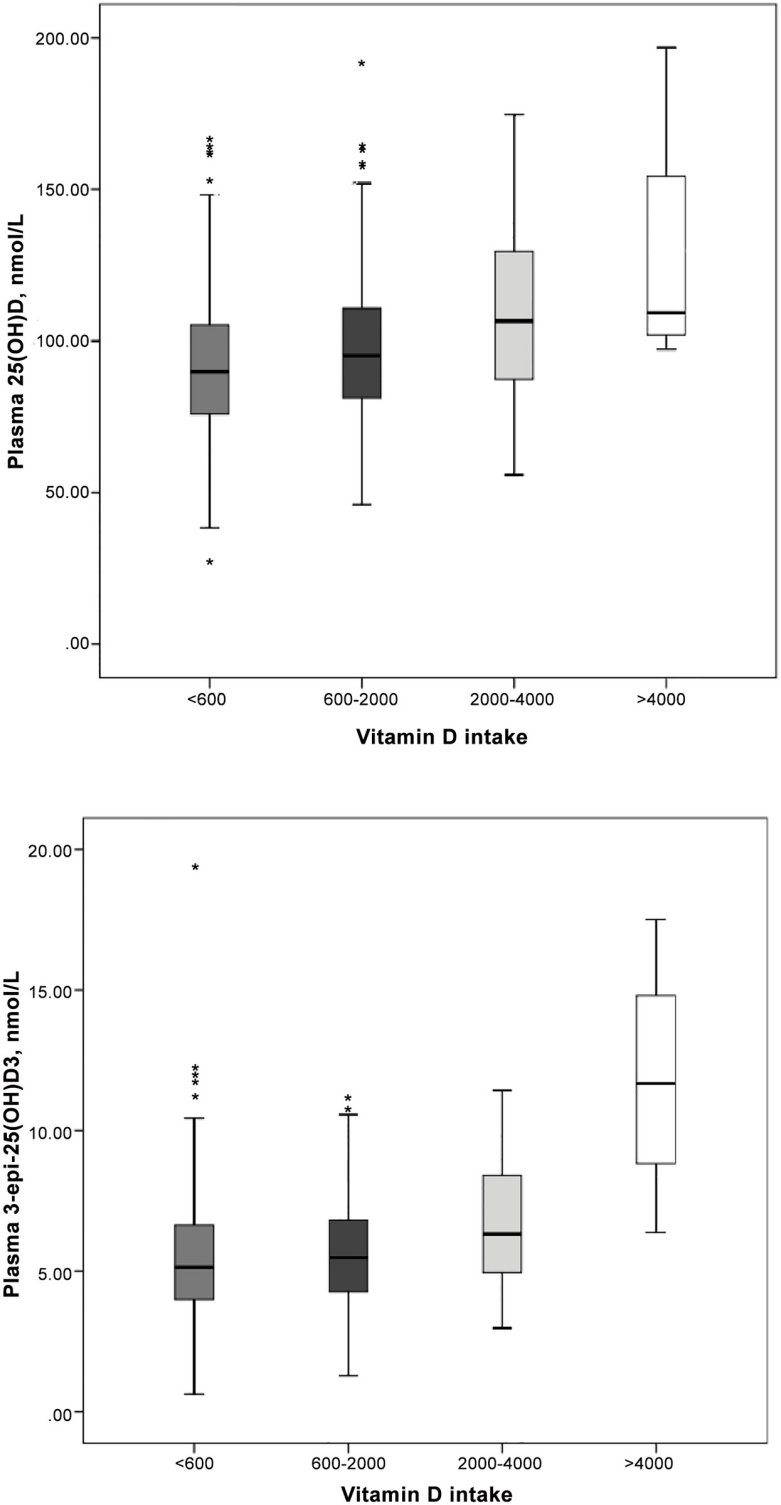
Absolute concentrations of (A) plasma 25(OH)D, and (B) plasma 3-epi-25(OH)D_3_ versus maternal vitamin D intake (IU/day) in a longitudinal cohort of pregnant women and their infants in Alberta, Canada. Black horizontal lines in each box represent medians; edges of each box represent 25^th^ and 75^th^ percentiles; the lines extending from each box represent 1.5 times the interquartile range; outliers are shown as stars.

**Association between women’s plasma 25(OH)D and 3-epi-25(OH)D**_**3**_ **concentration and maternal reported vitamin D intake**. The relationship between 25(OH)D and 3-epi-25(OH)D_3_ and vitamin D intake are shown in Fig 2a and 2b. There was a significant association between maternal plasma 25(OH)D and total reported vitamin D intake during the second trimester of pregnancy (β: 0.09, 95% CI: 0.06 to 0.13) in an adjusted linear regression model. Season of sampling and race were also significantly associated with 25(OH)D concentration (Table 4).

**Table 4:** Multiple Regression Model for 25(OH)D_3_, 3-epi-25(OH)D_3_, and %3-epi-25(OH)D_3_ in a Longitudinal Cohort of Pregnant Women and Their Infants in Alberta, Canada.

	25(OH)D^a^, β(95% CI)	P	3-epi-25(OH)D_3_^a^, β(95% CI)	P
Vitamin D intake	0.09 (0.4, 0.14)	<0.0001	0.13 (0.07, 0.18)	<0.0001
Season^b^	0.09 (0.04, 0.14)	<0.0001	0.03 (-0.05, 0.11)	0.4
Age	0.010 (-0.01, 0.01)	0.8	-0.01 (-0.01, 0.01)	0.8
BMI	-0.01 (-0.01, 0.01)	0.4	-0.01 (-0.02, -0.01)	0.03
Race^c^	0.12 (0.05, 0.20)	0.001	0.14 (0.01, 0.2)	0.01

^a^nmol/L.

^b^Summer (May 01-October 31) *vs*. winter (November 01-April 30).

^c^Caucasian *vs*. non-Caucasian.

In addition, the association between maternal plasma 3-epi-25(OH)D_3_ and total reported vitamin D intake during the second trimester was significant (β: 0.13, 95% CI: 0.07 to 0.18), as well as maternal BMI at second trimester and race in an adjusted linear regression model (Table 4). Plasma 3-epi-25(OH)D_3_ was significantly associated with reported vitamin D intake from supplements but not diet (data not shown). Women with plasma concentration of <75 nmol/L reported consuming less vitamin D (diet + supplements) compared to those with plasma concentration ≥ 75 nmol/L (median (IQR): 492 (320) IU/day *vs*. 622 (540) IU/day). In addition, when plasma levels were dichotomized at <50 *vs*. ≥ 50 and <75 *vs*. ≥ 75 nmol/L in a multiple logistic regression model, higher reported intake of vitamin D was associated with greater odds of vitamin D sufficiency during the second trimester within both ranges (OR: 2.17, 95% CI: 1.48 to 3.16 for ≥75 nmol/L and OR: 3.21, 95% CI: 1.17 to 8.83 for ≥50 nmol/L). Maternal age, BMI, season, and ethnicity were not significant effect modifiers or confounders in the model (data not shown).

## Discussion

To our knowledge, this is the largest study to date to show 44% of pregnant women with a higher socioeconomic status reported vitamin D intake (diet and supplements) not meeting the dietary reference intakes (DRI) recomendations. Using LC-MS/MS, we separated epimer from estimation of vitamin D status and were able to accurately estimate the prevalence of vitamin D insufficiency in our pregnant women. We also found that 20% of pregnant women had vitamin D concentraion <75 nmol/L, despite more than half of these women reporting taking more than 600 IU/day of vitamin D (the current RDA) based on their reported intake from diet and supplements.

In Northern countries such as Canada, the latitude of the country further reduces the potential contribution of dermal synthesis to the maintenance of appropriate vitamin D concentrations, placing women at greater risk for vitamin D deficiency/ insufficiency [21, 22]. There are few natural sources of vitamin D in the food supply: primarily fatty fish, beef liver, and egg yolk [7]. In North America, the main dietary source of vitamin D is through fortified dairy and dietary supplements [23, 24]. Canadian Community Nutrition Intakes data for non-pregnant women of child-bearing age (19–50 years) reported that 90% of Canadian women are not consuming the current recommendations for vitamin D through their diet [25]. In the current study, we showed that pregnant women are taking small amounts of vitamin D from food and beverages in their diet (<200 IU/day), and they are relying on supplemental sources of vitamin D to meet the current DRIs. We showed consuming lower doses of vitamin D (< than the current DRI) is associated with a higher rate of vitamin D insufficiency (>75nmol/L). Our findings are supportive of a growing body of literature suggesting that the recommended dose of vitamin D during pregnancy is inadequate in preventing vitamin D deficiency/insufficiency [26]. A recent study of maternal vitamin D status at third trimester of pregnancy and postpartum periods of 467 pregnant women in Toronto showed persistent vitamin D deficiency/ insufficiency in two-thirds of participants despite taking 400 IU/day of vitamin D through their prenatal vitamin D [27]. Another recent American longitudinal study of 193 pregnant women from mid-pregnancy to birth showed consuming a prenatal vitamin D supplement (334 IU/day) did not prevent precipitous drops in maternal plasma 25(OH)D concentration [28]. In fact, randomized clinical trials of 2000 and 4000 IU/day vitamin D during pregnancy have shown that vitamin D supplementation of 4000 IU/day was more effective in achieving serum concentrations ≥80 nmol/L of vitamin D [9]. One strength of the current study is its use of a quantification method capable of accurately measuring 25(OH)D (separating epimer) and adding the potential contribution of dietary vitamin D to the total intake estimate.

In addition, we demonstrated the presence of 3-epi-25(OH)D_3_ in all our pregnant women’s plasma samples across pregnancy (second trimester), and that including this epimer in the measurement of vitamin D status significantly influenced the classification of prevalence of vitamin D sufficiency in pregnancy. This finding is consistent with our previous study showing epimer can alter vitamin D status estimation of pregnant women at the time of delivery and in their cord blood [13]. Although previous studies have shown factors like age, season, and vitamin D supplementation affect 3-epi-25(OH)D_3_ [29, 30], in our study the only factor positively correlated with level of the 3-epi-25(OH)D_3_ was vitamin D intake. The fact that season significantly affected 25(OH)D concentration but not 3-epi-25(OH)D_3_ suggests that vitamin D supplements could be a source for 3-epi-25(OH)D_3_ as shown in a previous study [30]. Although the physiological role of the C_3_-epimerization pathway has not yet been elucidated, there are indications that it has some anti-proliferative activity and differentiation activity only at levels of approximately 10%-30%, respectively, compared to non-epimeric vitamin D metabolites [11, 12]. This has led to the suggestion that the C_3_-epimer should not be included in calculating 25(OH)D concentrations in serum. Clinical data has indicated that 9% of infants and 3% of adults would be misclassified as sufficient if the epimer had not been identified in the quantification [11]. These findings emphasize the need for further identification of the functional role and clinical significance of 3-epi-25(OH)D_3_ in the assessment of vitamin D status in pregnant women, given the fact that the cut-off for vitamin D sufficiency in pregnancy is unclear.

One of our study’s limitations is that the cohort was skewed toward women with annual income >$70,000 (2014 Canadian dollars), which could influence the generalizability of study findings to the entire population. In our study, only 20% of women had 25(OH)D <75 nmol/L, which is much lower than what is reported in other Canadian studies. The Canadian Health Measures Survey (CHMS) found that 63% of non-pregnant women aged 20–39 years had 25(OH)D <75 nmol/L [8], and a re-analysis of CHMS data, with standardization of the assay methodology, showed that 40% of women aged 19–30 years and 36% of women aged 31–50 years had 25(OH)D <50 nmol/L [31]. A study from Toronto showed that 65% of pregnant women had 25(OH)D <75 nmol/L and 31% had 25(OH)D <50 nmol/L [27]. Similarly, a study from Vancouver found that 65% of pregnant women had 25(OH)D <75 nmol/L and 24% had 25(OH)D <50 nmol/L [32]. A recent study showed that IOM recommendation is probably too low to achive 50 nmol/L for 97.5% of the populataion, although we did not show that in our study [33]. The lower rate of vitamin D insufficiency in our study could be related to the cohort characteristics involving very educated, high socio-economic status pregnant women. Still, we found a wide distribution of vitamin D intake and status, which allowed us to study the association between maternal reported vitamin D intake and maternal vitamin D concentrations. Furthermore, estimates from the 24-hour recall tool may be susceptible to usual dietary misreporting, involving inaccurate food descriptions and portion size estimation. However, given that vitamin D from foods in Canada makes such a small overall contribution to vitamin D dietary intake (Table 3), the accuracy of the 24-hour recalls is less critical in estimating women’s total vitamin D intake.

Another limitation of our study is the lack of information on sun exposure, skin color, and clothing coverage of participants. We used data on season as a proxy for sun exposure in our study. Studies [34, 35] have shown that ultraviolet radiation (UVR)-derived vitamin D is an important factor in 25(OH)D_3_ concentration. A Canadian study [36] of young adults found seasonal differences in 25(OH)D concentraions. Recent sun exposure predicted 25(OH)D concentration in the fall. The fall 25(OH)D concentration explained 45% of variation in 25(OH)D concentration in winter. Thus, it appears that although winter UVR exposure may be insufficient, variations in summer sun exposure will alter fall 25(OH)D concentration and thus indirectly influence winter vitamin D levels. Another recent Canadian study [35] showed summer sun exposure was a predictor of 25(OH)D_3_ concentration but fall sun exposure was not, which may be related to lower vitamin D-weighted climatological UV in Toronto during the fall. Although Calgary averages the greatest number of days/year with sunlight in Canada, study of adult participants in Calgary found a modest edffect of season on 25(OH)D concentration and serum PTH [37]. We showed that season of sampling and race were significantly associated with 25(OH)D concentration. While the current Canadian Cancer Society recommendations [38] are to avoid sun exposure or failing that, use of sunscreen at all times when out in the sun, our participants undoubtedly were getting sunlight exposure as seen by a seasonal variation in 25(OH)D.

In conclusion, we found a 20% rate of vitamin D insufficiency (<75 nmol/L) in a large, well-nourished cohort of pregnant women. Fifty percent of women who were classified as vitamin D insufficient (<75 nmol/L) consumed the RDA from both diet and supplements. This finding extends the literature suggesting that 600 IU/day may not be enough to achieve vitamin D status >75 nmol/L in pregnant women residing in higher latitudes with limited sun exposure. This cohort provided a vehicle for exploring this association, and problems noted in this best-case scenario group of educated and health-conscious women suggest the importance of interventions aimed at all Canadian pregnant women. These findings, together with existing data, highlight the need for re-evaluation of our current vitamin D recommendations for pregnant women residing in countries with higher latitude.

## Abbreviations

25(OH)D_2_: 25-hydroxyergocalciferol
25(OH)D_3_: 25-hydroxycholecalciferol
3-epi-25(OH)D_3_: 3-epi-25-hydroxycholecalciferol
LC-MS/MS: Liquid chromatography-tandem mass spectrometry
APrON: Alberta Pregnancy Outcomes and Nutrition

## References

[1] Aghajafari F, Nagulesapillai T, Ronksley PE, Tough SC, O’Beirne M, Rabi D. Association between maternal serum 25-hydroxyvitamin D level and pregnancy and neonatal outcomes: systematic review and meta-analysis of observational studies. BMJ. 2013 3 26;346:f1169 10.1136/bmj.f1169 23533188

[2] Thorne-Lyman A, Fawzi WW. Vitamin D during pregnancy and maternal, neonatal and infant health outcomes: a systematic review and meta-analysis. Paediat Perinat Ep. 2012;26:Suppl-90. 10.1111/j.1365-3016.2012.01283.x PMC3843348 22742603

[3] Canada Health [Internet]. Vitamin D and calcium: updated dietary reference intakes. Government of Canada [date modified 22 3 2012]. Available: http://www.hc-sc.gc.ca/fn-an/nutrition/vitamin/vita-d-eng.php.

[4] Institute of Medicine. Dietary reference intakes for calcium and vitmain D. Washington, DC: The National Academies Press; 2012.

[5] Hanley DA, Cranney A, Jones G, Whiting SJ, Leslie WD, Cole DE, et al Guidelines Committee of the Scientific Advisory Council of Osteoporosis Canada. Vitamin D in adult health and disease: a review and guideline statement from Osteoporosis Canada. CMAJ. 2010;182:E610–8. 20624865 10.1503/cmaj.091062 PMC2934797

[6] Holick MF, Binkley NC, Bischoff-Ferrari HA, Gordon CM, Hanley DA, Heaney RP, et al Evaluation, treatment, and prevention of vitamin D deficiency: an Endocrine Society clinical practice guideline. J Clin Endocrinol Metab. 2011;96:1911–30. 21646368 10.1210/jc.2011-0385

[7] Holick M. Vitamin D deficiency. N Engl J Med. 2007;357:266–81. 17634462 10.1056/NEJMra070553

[8] Whiting SJ, Langlois KA, Vatanparast H, Greene-Finestone LS. The vitamin D status of Canadians relative to the 2011 Dietary Reference Intakes: an examination in children and adults with and without supplement use. Am J Clin Nutr. 2011;94(1):128–35. 10.3945/ajcn.111.013268 21593503

[9] Hollis BW, Johnson D, Hulsey TC, Ebeling M, Wagner CL. Vitamin D supplementation during pregnancy: double-blind, randomized clinical trial of safety and effectiveness. J Bone Miner Res. 2011;26:2341–57. 10.1002/jbmr.463 21706518 PMC3183324

[10] Harvey NC, Holroyd C, Ntani G, Javaid K, Cooper P, Moon R, et al Vitamin D supplementation in pregnancy: a systematic review. Health Technol Assess. 2014;18(45):1–190. 10.3310/hta18450 25025896 PMC4124722

[11] Bailey D, Veljkovic K, Yazdanpanah M, Adeli K. Analytical measurement and clinical relevance of vitamin D(3) C3-epimer. Clin Biochem. 2013;46:190–6. 10.1016/j.clinbiochem.2012.10.037 23153571

[12] van den Ouweland JM, Beijers AM, Demacker PN, van Daal H. Measurement of 25-OH-vitamin D in human serum using liquid chromatography tandem-mass spectrometry with comparison to radioimmunoassay and automated immunoassay. J Chromatogr B Analyt Technol Biomed Life Sci. 2010;878:1163–8. 10.1016/j.jchromb.2010.03.035 20381436

[13] Aghajafari F, Field CJ, Rabi D, Kaplan BJ, Maggiore JA, O’Beirne M. Plasma 3-epi-25-hydroxycholecalciferol can alter the assessment of vitamin D status using the current reference ranges for pregnant women and their newborns. J Nutr. 2015; Pii:Jn220095. 10.3945/jn.115.220095 26609169

[14] Kaplan BJ, Giesbrecht GF, Leung BM, Field CJ, Dewey D, Bell RC, et al The Alberta Outcomes and Nutrition (APrON) cohort study: rationale and methods. Matern Child Nutr. 2014;10;44–60. 22805165 10.1111/j.1740-8709.2012.00433.x PMC6860282

[15] Gómez MF, Field CJ, Olstad DL, Loehr S, Ramage S, McCargar LJ, et al Use of micronutrient supplements among pregnant women in Alberta: results from the Alberta Pregnancy Outcomes and Nutrition (APrON) cohort. Matern Child Nutr. 2013;11:497–510. 10.1111/mcn.12038 23557540 PMC6860184

[16] Harville EW, Schramm M, Watt-Morse M, Chantala K, Anderson JJ, Hertz-Picciotto I. Calcium intake during pregnancy among white and African-American pregnant women in the United States. J Am Coll Nutr. 2004:23(1):43–50. 14963052 10.1080/07315724.2004.10719341

[17] Oken E, Ning Y, Rifas-Shiman SL, Rich-Edwards JW, Olsen SF, Gillman MW. Diet during pregnancy and risk of preeclampsia or gestational hypertension. Ann Epidemiol. 2007:17(9): 663–68. 17521921 10.1016/j.annepidem.2007.03.003 PMC2532559

[18] Rifas-Shiman SL, Rich-Edwards JW, Willett WC, Kleinman KP, Oken E, Gillman MW. Changes in dietary intake from the first to the second trimester of pregnancy. Pediatr Perinat Epidemiol. 2006:20(1):35–42. 10.1111/j.1365-3016.2006.00691.x PMC1488723 16420339

[19] Mannion CA, Gray-McDonald K, Johnson-Down L, Koski KG. Lactating women restricting milk are low on select nutrients. J Am Coll Nutr. 2007:26:2, 149–55. 17536126 10.1080/07315724.2007.10719596

[20] Haugen M, Brantsæter AL, Alexander J, Meltzer HM. Dietary supplements contribute substantially to the total nutrient intake in pregnant Norwegian women. Ann Nutr Metab. 2008:52: 272–80. 10.1159/000146274 18645244 PMC2813797

[21] Holick MF. High prevalence of vitamin D inadequacy and implications for health. Mayo Clin Proc. 2006;81:353–73. 16529140 10.4065/81.3.353

[22] Lips P. Vitamin D physiology. Prog Biophys Mol Biol. 2006;92:4–8. 16563471 10.1016/j.pbiomolbio.2006.02.016

[23] FDA (Food and Drug Administration). Agency Information Collection Activities; Submission for Office of Management and Budget Review; Comment Request; Food Labeling Regulations. Federal Register 2009;74(201)53743–6.

[24] Yetley E A. Assessing the vitamin D status of the US population. Am J Clin Nutr. 2008;88(2):558s–64S. 18689402 10.1093/ajcn/88.2.558S

[25] Canada Health [CD]. Canadian Community Health Survey Cycle 2.2 (2004). Nutrient intakes from food: Provincial, regional and national summary data tables, volume 1, 2 and 3.

[26] Wagner CL, Taylor SN, Johnson DD, Hollis BW. The role of vitamin D in pregnancy and lactation: emerging concepts. Womens Health (Lond Engl). 2012 5;8(3):323–40. 10.2217/whe.12.17 22554179 PMC4365424

[27] Kramer CK, Ye C, Swaminathan B, Hanley AJ, Connelley PW, Sermer M, et al The persistence of maternal vitamin D deficiency and insufficiency during pregnancy and lactation irrespective of season and supplementation. Clin Endocrinol (Oxf). 2016 5;84(5):680–6. 10.1111/cen.12989 26641010

[28] Ozias MK, Kerling EH, Christifano DN, Scholtz SA, Colombo J, et al Typical prenatal vitamin D supplement intake does not prevent decrease of plasma 25-hydroxyvitmainD at birth. J Am Coll Nutr. 2014;33(5):394–9. 10.1080/07315724.2013.879843 25302772 PMC4224609

[29] Cashman KD, Kinsella M, Walton J, Flynn A, Hayes A, Lucey AJ, et al The 3 epimer of 25-hydroxycholecalciferol is present in the circulation of the majority of adults in a nationally representative sample and has endogenous origins. J Nutr. 2014 7;144(7):1050–7. 10.3945/jn.114.192419 24828024 PMC4056645

[30] Bailey D, Perumal N, Yazdanpanah M, Al Mahmud A, Baqui AH, Adeli K, et al Maternal-fetal-infant dynamic of C3 epimer of 25-hydroxy vitamin D. Clin Biochem. 2014;47:816–22. 10.1016/j.clinbiochem.2014.01.015 24462965

[31] Sarafin K, Durazo-Arvizu R, Tian L, Phinney KW, Tai S, Camara JE, et al Standardizing 25-hydroxyvitamin D values from the Canadian Health Measures Survey. Am J Clin Nutr. 2015 11;102(5):1044–50. 10.3945/ajcn.114.103689 26423385 PMC4625585

[32] Wangyang L, Green TJ, Innis SM, Barr SI, Whiting SJ, Shand A, et al Suboptimal vitamin D levels in pregnant women despite supplemental use. Can J Public Health. 2011;102(4);308–12. 21913590 10.1007/BF03404056 PMC6974126

[33] Veugelers PJ, Pham TM, Ekwaru JP. Optimal vitamin D supplementation doses that minimize the risk for both low and high serum 25-hydroxyvitamin D concentrations in the general population. Nutrients. 2015 12 4;7(12):10189–208. 10.3390/nu7125527 26690210 PMC4690079

[34] Lucas RM, Ponsonby AL, Dear K, Valery PC, Taylor B, van der Mei I, et al Vitamin D status: multifactorial contribution of environment, genes and other factors in healthy Australian adults across a latitude gradient. J Steroid Biochem Mol Biol. 2013 7;136:300–8. 10.1016/j.jsbmb.2013.01.011 23395985

[35] Sham L, Yeh EA, Mefalhaes S, Parra EJ, Gozdzik A, Banwell B, et al Evaluation of fall Sun Exposure Score in predicting vitamin D status in young Canadian adults, and the influence of ancestry. J Photochem Photobiol B. 2015 4;145:25–2. 10.1016/j.jphotobiol.2015.02.007 25752862

[36] Gozdzik A, Barta JL, Weir A, Cole DEC, Vieth R, Whiting SJ, et al Serum 25-hydroxyvitamin D concentrations fluctuate seasonally in young adults of divers ancestry living in Toronto. J Nutr. 2010;140:2213–20. 10.3945/jn.110.126284 20980651

[37] Rucker D, Allan JA, Fick GH, Hanley DA. Vitamin D insufficiency in a population of healthy western Canadians. CMAJ. 2002 6 11;166(12):1517–24. 12074117 PMC113796

[38] Canadian Cancer Society [Internet]. Being safe in the sun. 2016 Available: http://www.cancer.ca/en/prevention-and-screening/live-well/sun-and-uv/being-safe-in-the-sun.

